# Effects of *Flammulina velutipes* mushroom residues on growth performance, apparent digestibility, serum biochemical indicators, rumen fermentation and microbial of Guizhou black goat

**DOI:** 10.3389/fmicb.2024.1347853

**Published:** 2024-01-24

**Authors:** Yong Long, Wen Xiao, Yanpin Zhao, Chao Yuan, Defeng Wang, Yang Yang, Chaozhi Su, Pramote Paengkoum, Yong Han

**Affiliations:** ^1^Guizhou University of Traditional Chinese Medicine, Guiyang, China; ^2^Institute of Animal Husbandry and Veterinary Sciences, Guizhou Academy of Agricultural Sciences, Guiyang, China; ^3^Key Laboratory of Animal Genetics, Breeding and Reproduction in the Plateau Mountainous Region, Ministry of Education, Guizhou University, Guiyang, China; ^4^School of Animal Technology and Innovation, Institute of Agricultural Technology, Suranaree University of Technology, Nakhon Ratchasima, Thailand

**Keywords:** apparent digestibility, growth performance, rumen fermentation, rumen microorganisms, serum biochemical indicators

## Abstract

**Introduction:**

The primary objective of the current study was to evaluate the effects of *Flammulina velutipes* mushroom residue (FVMR) in a fermented total mixed ration (FTMR) diet on the fattening effect and rumen microorganisms in Guizhou black male goats.

**Methods:**

A total of 22 Guizhou black male goats were allocated into two groups using the Randomized Complete Block Design (RCBD) experimental design. The average initial weight was 22.41 ± 0.90 kg and with 11 goats in each group. The control group (group I) was fed the traditional fermentation total mixed ration (FTMR) diet without FVMR. Group II was fed the 30% FVMR in the FTMR diet.

**Results:**

The results showed that compared with group I, the addition of FVMR in the goat diet could reduce the feed cost and feed conversion ratio (FCR) of group II (*p* < 0.01). Notably, the apparent digestibility of crude protein (CP), acid detergent fiber (ADF), neutral detergent fiber (NDF), and dry matter (DM) were higher in group II (*p* < 0.01). The levels of growth hormone (GH), immunoglobulin A (IgA), and immunoglobulin M (IgM) in group II were higher than that of group I (*p* < 0.01), which the level of glutamic oxalacetic transaminase (ALT) and interleukin-6 (IL-6) was noticeably lower than that of group I (*p* < 0.01). 30% FVMR in FTMR diets had no effect on rumen fermentation parameters and microbial composition at the phylum level of Guizhou black male goats (*p* > 0.05). However, at the genus level, the relative abundance of *bacteroidal_bs11_gut_group*, *Christensenellaceae_R-7_group* and *Desulfovibrio* in group II was lower than in group I (*p* < 0.05), and the relative abundance of *Lachnospiraceae_ND3007_group* was higher than in group I (*p* < 0.01).

**Discussion:**

In conclusion, the results of the current study indicated that 30% FVMR in the FTMR diet improves rumen fermentation and rumen microbial composition in Guizhou black male goats, which improves growth performance, apparent digestibility, and immunity.

## 1 Introduction

Mushroom residue is the main by-product left after the production of edible fungi, and its components are mainly wood chips, cottonseed hulls, corn cobs, rice straw, and sugarcane residue. For every kilogram of fresh edible mushroom produced, about 5 kilograms of mushroom residue products will be produced (Gao et al., 2021). In 2020, the output of mushroom residue in China reached 132–203 million tons. At present, the primary uses of mushroom residue are the reproduction of edible fungi, the development of animal feed, composting, the development of biomass energy, soil improvement, and restoration, sewage treatment, extraction of biologically active substances and raw materials for the food industry (Guo et al., 2022; Leong et al., 2022). The nutrients in the mushroom residue after harvesting edible fungi have not been completely degraded and the contents of crude protein (CP), ether extract (EE), crude fiber (*CF*), and amino acids are relatively high (Estrada et al., 2009). The mushroom sticks produce many fungal mycelium and beneficial bacteria during edible mushroom growth. During the growth process of mycelium, enzymatic hydrolysis can produce a variety of sugars (water-soluble dietary fiber), polyphenolic compounds (quercetin, catechin, gallate, caffeate, etc.), compounds with anti-cancer activity (Flammulina velutipes polysaccharides, fungal immunomodulatory proteins, steroid compounds, monoterpenes, etc.) have lipid-lowering, antioxidant and anti-cancer effects on animals (Hertog et al., 1993; Yeh et al., 2014; Najafi et al., 2019). In addition, the characteristic mushroom fragrance of mycelia can improve the palatability of feed and stimulate the appetite of livestock (Koutrotsios et al., 2014). A previous study by Kim et al. (2011) indicated that 10% fermented oyster mushroom residue in the diet could enhance Holstein calves’ growth performance and blood biochemical indicators were not affected. Huang et al. (2023) found that the incorporation of 15% *Pleurotus eryngii* fermentation residues in Hu sheep diets had the best effect on improving production performance, rumen microbial composition, and rumen microbial abundance of Hu sheep. Mandale et al. (2023) considered that supplementing 10% mushroom residues in Berari goat diets could reduce feed costs and improve growth performance in Berari goats. Relevant studies have shown that the incorporation of FVMR in goat diets could enhance apparent digestibility, slaughter performance, nitrogen biological value, and nitrogen deposition rate. At the same time, the meat quality of goats was also significantly improved (Meng et al., 2016, 2017).

Guizhou black goat is one of the three typical goat breeds in Guizhou, China, which have strong survival ability due to the special ecological environment of karst landform and harsh natural selection. Guizhou black goat has always been famous for its high meat quality and low cholesterol. It has been listed as a local excellent varieties protection list in Guizhou Province. It has the characteristics of excellent meat quality, good gregariousness, wide feeding range, walking ability, and strong stress resistance, and an annual inventory of about 500,000.

In current research, there are relatively few research reports on the application of mushroom residue as feed in animal production and research reports on feeding goats with FVMR are even rarer. In the previous single-cage feeding study, our research group concluded that the chemical composition of FVMR is: dry matter (92.67%), crude protein (10.83%), crude ash (10.94%), neutral detergent fiber (53.79%), ether extract (2.79%), calcium (3.19%), and phosphorus (0.43%). The incorporation of FVMR (30, 40, 50, 60%) in diets through fermentation treatment could change the feed quality, and affect the feeding behavior, rumination behavior and diarrhea rate of Guizhou black male goats. Finally, the 30% FVMR in fermented total mixed diet (FTMR) group had the best effect in improving the feed quality and feeding behavior of Guizhou black male goats, promoting rumination activity, and reducing diarrhea rates (Long et al., 2022). It was hypothesized that long-term feeding of 30% FVMR in FTMR diets may have many positive effects on the production performance, rumen fermentation, serum biochemical indicators, and rumen microorganisms of Guizhou black male goats. Therefore, the objective of the current study was to conduct a more comprehensive explanation on the effects of 30% FVMR in FTMR diets on the production performance, rumen fermentation, serum biochemical indicators, and rumen microorganisms of Guizhou black male goats. In addition, FVMR may ultimately achieve the goal of reducing feeding costs by replacing some of the more expensive roughage.

## 2 Materials and methods

### 2.1 Animal ethics statement

The experiment was conducted at the Maiping Experimental Base of the Guizhou Provincial Institute of Animal Husbandry and Veterinary Medicine. All animals were meticulously conducted in accordance with animal welfare guidelines and were subject to stringent regulatory oversight by the Experimental Animal Ethics Committee of Guizhou University in Guizhou, China (EAE-GZU-2021-E024).

### 2.2 Materials, animals, diets, and experimental design

*Flammulina velutipes* mushroom residue (The dregs left after the *Flammulina velutipes* mushroom are picked after about 10 days of growth. At this time, the length of the *Flammulina velutipes* mushroom residue was about 15–20 cm.): was provided by Xuerong Biotechnology Co., Ltd. (No. 2 *Flammulina velutipes* Factory) (Bijie, China). White-rot fungi were purchased from the Beijing Microbiological Culture Collection Center (Beijing, China). The mixed fermentation agent (lactic acid bacteria; yeast; *Bacillus licheniformis*; *Enterococcus faecalis*; *Bacillus subtilis*; etc., number of viable bacteria ≥5 × 1,011 CFU/g) was acquired from Luoyang Oukebaik Biotechnology Co., Ltd. (Luoyang, China). The mixed enzyme preparation (Contains cellulase ≥7,000 U/g, β-glucanase ≥12,000 U/g, xylanase ≥30,000 U/g, and pectinase ≥3,000 U/g) was acquired from CJ Youtel Biotechnology Co., Ltd. (Shanghai, China).

The FTMR was reconfigured according to the optimal FVMR addition ratio formulation determined in previous research by Long et al. (2023a). Twenty-two healthy, 5–6 months old, Guizhou black male goats with 22.41 ± 0.90 kg live body weights were used in this experiment. The goats were allocated into two groups using the Randomized Complete Block Design (RCBD) experimental design, with each group consisting of 11 test duplicates. The control group (group I) was fed with traditional FTMR without FVMR according to the feed formula. The fed group II was made into FTMR by adding 30% FVMR according to the designed formula and adding white-rot fungi, mixed starter, and mixed enzyme preparation according to the product instructions and mixed evenly, adjusting the moisture content to 42%, put it into fermentation bags (70 cm × 130 cm) (Guizhou Weilai Technology Co., Ltd. Guiyang, China) for room temperature fermentation and seal for 15 days. In the feed quality testing test, the same method was used but smaller fermentation bags (35 cm × 50 cm) were used to produce FTMR. A total of 12 fermentation bags were prepared, with 6 replicates in each group. The entire feeding experimental timeline spanned 75 days, comprising a 15-day pre-trial period and a 60-day period dedicated to the collection of data and samples. The composition and nutritional levels of the basic diet formulated according to NRC (2007) nutritional requirements are shown in Table 1.

**Table 1:** Composition and nutrient levels of diets.

Item	Group	
	I	II
Ingredients (DM)		
Corn	28.00	21.50
Wheat bran	7.00	5.00
Rice bran	9.10	10.00
Soybean meal 43	4.50	5.00
Rapeseed meal	3.00	3.38
NaCl	0.50	0.51
1% composite premix^a^	0.46	0.50
Urea	1.64	1.11
Cane molasses	–	3.00
Peanut vine	45.80	20.00
*Flammulina velutipes* mushroom residue	–	30.00
Total	100.00	100.00
Chemical composition^b^(Air-dry basis)		
Dry matter (DM)	80.73	75.59
Neutral detergent fiber (NDF)	39.56	36.14
Crude protein (CP)	20.82	19.08
Ether extract (EE)	3.09	4.47
Calcium (Ca)	0.58	1.36
Total phosphorus (P)	0.50	0.71
Metabolizable energy (ME), MJ/kg	11.61	10.32

I, Traditional FTMR without FVMR diets; II, 30% FVMR in FTMR diets.

^a^ Premix provides per kg of ration: Vitamin A 10,000 IU, Vitamin D 15,000 IU, Vitamin E 200 IU, Iron 1,000–7,500 mg, Zinc 5,000–3,750 mg, Copper 140–150 mg, Iodine 5–200 mg, Manganese 600–3,000 mg, Cobalt 4–40 mg, Selenium 3.5–10 mg, calcium 4–20% and phosphorus 2–8%.

^b^ The chemical composition is measured value.

In this experiment, each goat was kept individually in a separate fully automatic precision-feeding metabolic cage. A total of 22 fully automatic precision-feeding metabolic cages were used in this experiment. The fully automatic precision feeding metabolic cage system consists of automatic weighing, automatic drinking water, automatic temperature, humidity sensing, hydrogen sulfide, and safety sensing systems. Each metabolic cage (2 m^2^) is made of stainless steel, and each cage was strictly sterilized before the experiment and vaccinated and dewormed following the farm’s standard operating procedures. During the pre-test and formal test phases, the diet proportion was the same for every test group. The test goats were fed on time at 9:00 and 17:00 every day. Before each feeding, ensure that the remaining feed content in the trough during the second feeding is approximately 10% of the previous feed.

### 2.3 Growth performance and economic benefits

During the experiment, the remaining feed was cleaned before each feeding. In addition, the fully automatic precision feeding system automatically transmitted the average daily gain (ADG) of the goats to the computer at regular intervals every day. The experimenter recorded the weight of the remaining feed before each feeding and calculated the dry matter intake (DMI), feed conversion ratio (FCR), feed weight gain cost and weight gain benefit according to the method of Long et al. (2022).

### 2.4 Sample collection and apparent digestibility

The duration of each digestibility trial was 12 days, including 7 days of adaption and 5 days of total collection of feces. Before the morning feeding from day 70 to 75, the total feces of each male goat were collected, weighed, and recorded. The manure samples were combined in equal proportions, at a ratio of 20% relative to the weight of fresh manure. Subsequently, the nitrogen was then stabilized by adding 10% diluted sulfuric acid, and the samples were kept in a refrigerator at −20°C (Li et al., 2022).

The drying, crushing, sieving, and preservation processes of feed and feces samples were carried out according to the method of Yang et al. (2018). The dried samples were utilized for the analysis of various components, including dry matter (DM, method No. 930.15), crude protein (CP, method No. 976.05), calcium and phosphorus (method 935.13), ether extract (EE, method No. 973.18) according to the Association of official Analytical Chemists (AOAC, 2000). Neutral detergent fiber (NDF) in feed was determined according to the method of Van Soest et al. (1991) without thermostable α-amylase but uses sodium sulfite and NDF is expressed without residual ash. The endogenous indicator acid-insoluble ash (AIA) was used to calculate apparent nutrient digestibility (The method was to quantify digestibility) according to the method of Van Keulen and Young (1977).

### 2.5 Rumen fluid collection, rumen fermentation parameters, and rumen microbial testing

On the morning of the last day of the experiment, 2 h after feeding, six goats were randomly selected from the 11 goats in each group to collect rumen fluid using a negative-pressure sampler, with the tube inserted orally via the esophagus into the mid-rumen. To prevent contamination, the rumen fluid collected initially was discarded. About 50 mL of rumen fluid was collected from each goat. The rumen fluid was filtered through 4 layers of gauze, and the pH (PHS-3E, Shanghai Leici Instrument Co., Ltd., Shanghai, China) value of the rumen fluid was detected immediately. The concentration of NH_3_-N was measured by a microplate reader (ELX800, BioTek Instrument Co., Ltd., USA) following the method of Paengkoum and Han (2009).

Gas chromatography (GC) was employed to detect VFA. Take an appropriate amount of rumen fluid sample and place it in a 2 mL centrifuge tube. Samples were extracted in 50 μL of 15% phosphoric acid with 100 μL of 125 μg/mL 4-methyl valeric acid solution as IS (The IS was used to correct for injection variability between samples and minor changes in the instrument response) and 400 μL ether. Subsequently, the samples were centrifuged at 4°C for 10 min at 12000 rpm after vortexing for 1 min, and the supernatant was transferred into the vial before GC–MS analysis (Han et al., 2018).

The GC–MS analysis was performed on a trace 1,300 gas chromatograph (Thermo Fisher Scientific, USA). The GC–MS was fitted with a capillary column Agilent HP-INNOWAX (30 m × 0.25 mm ID × 0.25 μm), and helium was used as the carrier gas at 1 mL/min. The injection was made in split mode at 10:1 with an injection volume of 1 μL and an injector temperature of 250°C. The ion source and interface temperature were 300°C and 250°C, respectively. The column temperature was programmed to increase from an initial temperature of 90°C, followed by an increase to 120°C at 10°C/min, to 150°C at 5°C/min, and finally to 250°C at 25°C/min, which was maintained for 2 min (total run-time of 15 min). Mass spectrometric detection of metabolites was performed on ISQ 7000 (Thermo Fisher Scientific, USA) with electron impact ionization mode. Single ion monitoring (SIM) mode was used with an electron energy of 70 eV (Hsu et al., 2019; Zhang et al., 2019).

### 2.6 Serum biochemical indicators

At the end of the experiment, blood was collected from the jugular vein before feeding. 10 mL of blood was collected from each goat. The serum was separated by a low-speed centrifuge and stored in a − 20°C refrigerator until testing. Immunoglobulin M (IgM), Immunoglobulin G (IgG), Immunoglobulin A (IgA), Interleukin-2 (IL-2), Interleukin-6 (IL-6), Tumor Necrosis Factor-α (TNF-α), Interferon-gamma-γ (INF-γ), Glutamic oxalacetic transaminase (ALT), Alanine aminotransferase (AST), and Growth Hormone (GH) were measured with a microplate reader (ELX800, BioTek, USA) according to the detailed steps of the ELISA kit (Haoyuan Biotechnology Co., Ltd., Yibin, China).

### 2.7 Rumen microbiome detection and analysis

Rumen fluid samples were snap frozen and stored at −80°C after collection. Bacterial DNA was isolated from the Rumen fluid using a MagPure Soil DNA LQ Kit (D6356-02, Magen, Hilden, Germany) following the manufacturer’s instructions. DNA concentration and integrity were measured by a NanoDrop 2000 spectrophotometer (Thermo Fisher Scientific, Waltham, MA, USA) and agarose gel electrophoresis. The V3-V4 hypervariable region of the bacterial 16S rRNA gene was amplified by PCR using primers (343F, 5′-TACGRAGCAGCAG-3′, 798R: 5′-AGGGTATCTAATCCT-3′) in a 25 μL reaction. The resulting sample barcode was included in the reverse primer, and both primers were individually ligated to Illumina sequencing adapters.

Gelatin electrophoresis was used to visualize the Amplicon quality. Using Agencourt AMPure XP beads (Beckman Coulter Co., USA), PCR products were purified, and the Qubit dsDNA kit was used to quantify the results. Subsequently, the concentrations were adjusted to prepare for the sequencing process. Sequencing was carried out on an Illumina NovaSeq 6000 platform, employing two paired-end read cycles, each with a length of 250 bases. (Illumina Inc., San Diego, CA; OE Biotech Company, Shanghai, China).

The format of the raw sequencing data was FASTQ. Then the Cutadapt software was used to preprocess the obtained data, detect and cut off the joints. After completion, DADA2 and QIIME2 were used to filter the obtained data, denoise, merge, detect, and cut off chimeric reads. The methods are based on Callahan et al. (2016) and Bolyen et al. (2019) respectively. At last, the software outputs the representative reads and the ASV abundance table. The representative read of each ASV was selected using the QIIME2 package. All representative reads were annotated and blasted against Silva database Version 138 (or unite) (16 s/18 s/ITS rDNA) using q2-feature-classifier with the default parameters. The microbial diversity of rumen fluid samples was analyzed by alpha diversity analysis, and the Chao1, Simpson, and Shannon index values were calculated according to the methods of Hill et al. (2003) and Chao and Bunge (2002). The Unifrac distance matrix performed by QIIME software was used for unweighted Unifrac Principal coordinates analysis (PCoA) and phylogenetic tree construction. The 16S rRNA gene amplicon sequencing and analysis were conducted by OE Biotech Co., Ltd. (Shanghai, China).

### 2.8 Statistical analysis

All the original data obtained in this experiment were first sorted and recorded using Excel 2021. The data’s normal distribution was first ascertained using the Shapiro–Wilk test. Finally, the recorded data software was used for statistical analysis through SPSS 26.0. Data analyzes were one-way ANOVA and multi-covariate ANOVA with general linear model (GLM module). The Duncan’s test and the LSD method were used to conduct multiple comparisons and significant difference tests. All results were expressed as means and standard error of the mean (SEM). In the analysis, a significance level of *p* < 0.05 was regarded as statistically significant, and *p* < 0.01 as highly significant.

## 3 Results

### 3.1 Growth performance and apparent digestibility

The DMI, FCR, and feed weight gain cost of group I was higher than that of group II (*p* < 0.01). Compared with group I, the FBW (Average final body weight), TWG (Average total weight gain), and weight gain benefit of group II were increased, respectively, by 4.99, 10.89, and 10.82%, and each goat of group II earned 17.35 CNY (Chinese Yuan) more than the group I. There was no significant difference in ADG between the two groups; however, in comparison to Group I, the ADG of group II was also increased by 10.83% and the feed cost of group II was reduced by 25% (Table 2). In contrast to group I, the apparent digestibility of DM, CP, and NDF in group II were increased, by 13.84, 7.07, and 7.74% (*p* < 0.01). The apparent digestibility of EE did not differ between the two groups (*p* > 0.05) (Table 3).

**Table 2:** Effects of different diets on the performance and economic benefits of Guizhou black male goats.

Item	Group		SEM	*p*-value
	I	II		
DMI, kg/d	0.97^a^	0.86^b^	0.01	<0.01
IBW, kg	22.10	22.73	0.90	0.746
FBW, kg	30.27	31.78	0.97	0.462
TWG, kg/hd	8.17	9.06	0.30	0.143
ADG, g	136.18	150.93	4.95	0.143
FCR	7.14^a^	5.82^b^	0.20	<0.01
Live goat real-time price, CNY/kg ^a^	40	40	–	–
Feed cost, CNY/kg	2.72	2.04	–	–
Feed weight gain cost, CNY/kg	21.89^a^	16.44^b^	1.02	<0.01
Weight gain benefit, CNY/one	160.38	177.73	5.83	0.143

*p* < 0.05, significant; *p* < 0.01, extremely significant. SEM, standard error of the mean. Different superscript letters indicate significant differences. The data description method of this paper’s rest of the tables is the same.

I, Traditional FTMR without FVMR diets; II, 30% FVMR in FTMR diets.

^a^ Live goat real-time price and feed raw material prices (For example, the FVMR price is 120 CNY per ton) are the current price in Guizhou Province, China.

ADG = (end of test weight − beginning of test weight)/number of feeding days; FCR = DMI/ADG; DMI (kg/d) = [feeding amount (kg) × diet DM (%) − leftover (kg) × leftover DM (%)]/[number of black goats per group × number of trial days (d)]; Feed weight gain cost (CNY/kg) = total feed intake × feed unit price (CNY)/total weight gain; Weight gain benefit (CNY/one goat) = (real-time price of live black goat − feed weight gain cost) × total weight gain.

Kg/one goat, Kilograms per black goat; CNY, Chinese Yuan; CNY/kg, CNY per kg; CNY/one, CNY per black goat; IBW, Average initial body weight; FBW, Average final body weight; TWG, Average total weight gain; Feed weight gain cost, Feed weight gain cost assume cost of gaining 1 kg BW by each animal.

**Table 3:** Effects of diets on apparent digestibility of Guizhou black male goats (%).

Item	Group		SEM	*p*-value
	I	II		
DM	72.49^b^	82.52^a^	1.59	<0.01
EE	72.23	73.70	0.91	0.462
CP	75.29^b^	80.61^a^	0.95	<0.01
NDF	65.85^b^	70.95^a^	0.84	<0.01

*p* < 0.05, significant; *p* < 0.01, extremely significant. SEM, standard error of the mean. Different superscript letters indicate significant differences.

I, Traditional FTMR without FVMR diets; II, 30% FVMR in FTMR diets.

DM, dry matter; EE, ether extract; CP, crude protein; NDF, neutral detergent fiber; ADF, acid detergent fiber.

### 3.2 Rumen fermentation parameters

The pH level in Group II was higher than that in Group I (*p* < 0.05). NH_3_-N, TVFA (Total volatile fatty acids), and A/P were unaffected by the addition of FVMR (*p* > 0.05). The proportion of acetic acid in TVFA in group I was higher than that in group II (*p* < 0.01), while there was no difference in the proportions of propionic acid, butyric acid, isovaleric acid, isobutyric acid, valeric acid, and caproic acid in TVFA between the two groups (*p* > 0.05) (Table 4).

**Table 4:** Effects of different diets on ruminal fermentation parameters of Guizhou black male goats.

Item	Group		SEM	*p*-value
	I	II		
pH	6.34^b^	6.58^a^	0.06	0.030
NH_3_-N, mg/dL	12.31	12.76	0.93	0.832
TVFA, mmol/L	54.53	48.12	6.41	0.640
VFA,^a^ mol/100 mol				
Acetic acid	70.13^a^	67.93^b^	0.43	<0.01
Propionic acid	13.92	14.47	0.47	0.569
Butyric acid	11.87	13.50	0.58	0.166
Isobutyric acid	1.65	1.68	0.13	0.928
Isovaleric acid	1.21	1.26	0.10	0.810
Valeric acid	1.01	0.98	0.03	0.667
Caproic acid	0.20	0.17	0.02	0.376
A/P	5.20	4.79	0.27	0.460

*p* < 0.05, significant; *p* < 0.01, extremely significant. SEM, standard error of the mean. Different superscript letters indicate significant differences.

I, Traditional FTMR without FVMR diets; II, 30% FVMR in FTMR diets.

^a^ The data of each VFA individual in the table, respectively, represent the proportion of this individual in TVFA.

NH_3_-N, ammonia nitrogen; TVFA, total volatile fatty acids; VFA, volatile fatty acids; A/P, acetic acid / propionic acid.

### 3.3 Serum biochemical indicators

It can be depicted from Table 5 that adding FVMR to the feed has an impact on the serum biochemical indicators of black goats. The FVMR in the FTMR diet had no effect on IgG, IL-2, TFN-α, or IFN-γ (*p* > 0.05). Moreover, feeding black goats at a rate of FVFM in the FTMR resulted in higher serum IgG, TFN-α, and IFN-γ levels than group I. In addition, the level of IL-2 in group II was lower than that of group I. But there is no significance (*p* > 0.05). The GH, IgM, and IgA level in group II was higher than in group I (*p* < 0.01). FVFM in the FTMR to feed black goats could reduce serum IL-6 and ALT levels (*p* < 0.01), Similar, it could also reduce AST levels (*p* < 0.05) (Table 5).

**Table 5:** Effects of different diets on serum biochemical indexes of Guizhou black male goats.

Item	Group		SEM	*p*-value
	I	II		
GH, ng/mL	2.89^b^	4.13^a^	0.17	<0.01
IgG mg/mL	4.21	4.63	0.13	0.102
IgM mg/mL	1.01^b^	1.49^a^	0.06	<0.01
IgA μg/mL	208.44^b^	251.43^a^	7.17	<0.01
IL-2 pg/mL	943.64	909.84	35.72	0.644
IL-6 pg/mL	157.80^a^	133.35^b^	3.86	<0.01
TFN-α pg/mL	225.68	238.54	7.73	0.415
IFN-γ pg/mL	500.17	560.94	18.94	0.110
ALT pg./mL	671.02^a^	601.71^b^	11.41	<0.01
AST mmol/L	319.78^a^	285.82^b^	7.25	0.016

*p* < 0.05, significant; *p* < 0.01, extremely significant. SEM, standard error of the mean. Different superscript letters indicate significant differences.

I, Traditional FTMR without FVMR diets; II, 30% FVMR in FTMR diets.

IgM, Immunoglobulin M; IgA, Immunoglobulin A; IgG, Immunoglobulin G; IL-6, Interleukin-6; IL-2, Interleukin-2; TNF-α, Tumor Necrosis Factor-α; INF-γ, Interferon-gamma-γ; ALT, Glutamic oxalacetic transaminase; AST, Alanine aminotransferase.

### 3.4 Microbiota composition

**Figure 1:**
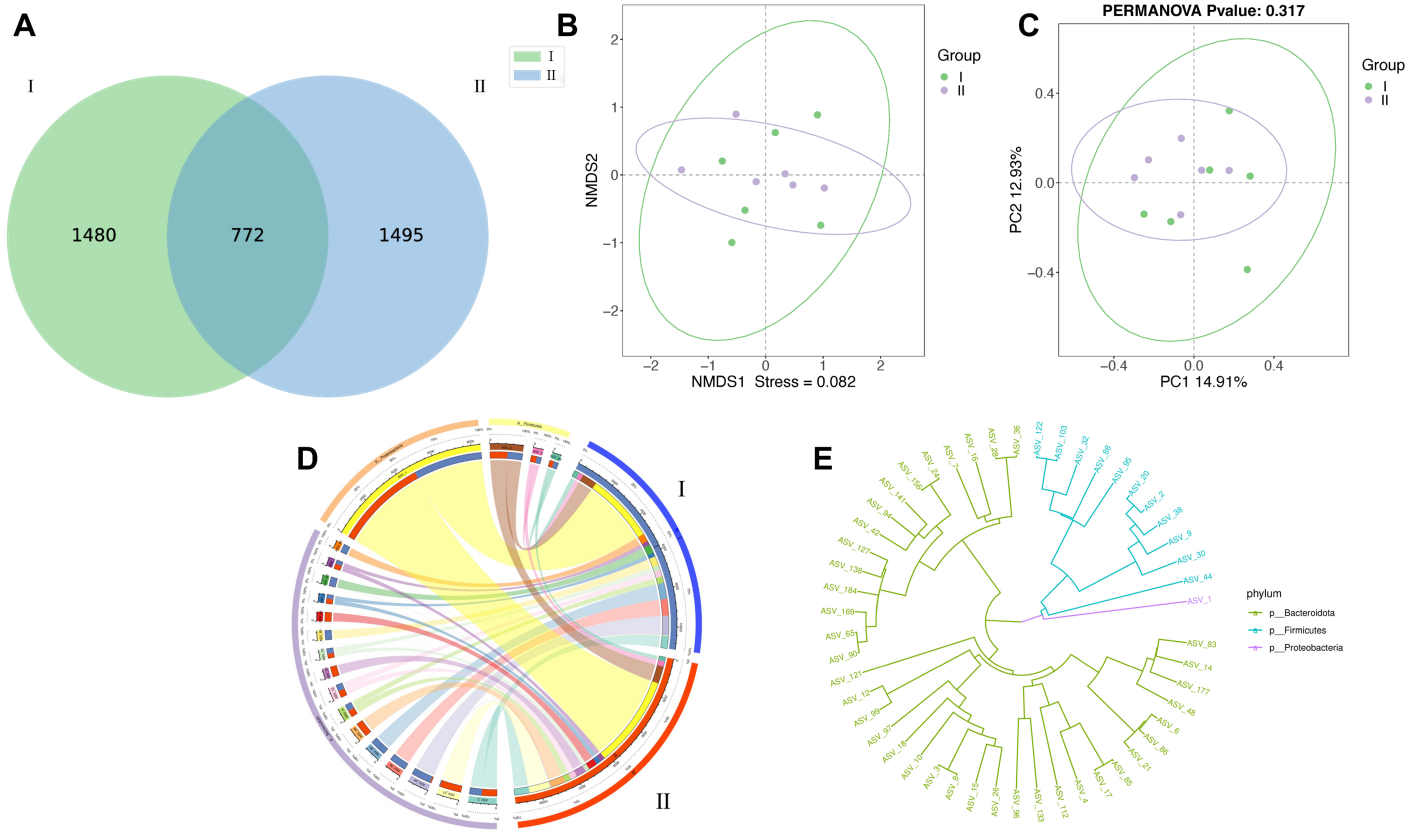
Effects of different diets on rumen microorganisms of Guizhou black male goats. **(A–E)** Are ASV-Venn, Principal coordinate analysis (PCoA) non-metric multidimensional scale analysis (NMDS), ASV-Circos, and ASV- Phylogenetic diagram analysis of I and II.

The results of the Venn analysis showed that at the ASV level, specific bacterial ASV accounted for 39.50% (1480) of the total ASV sequence number in group I, specific bacterial ASV accounted for 39.90% (1495) of the total ASV sequence number in group II (Figure 1A). In addition, the number of bacterial ASVs shared by groups I and II was 772 (22.22%). It was concluded by the PCoA (Figure 1B) and NMDS (Figure 1C) diagrams that the distribution of samples in group II is relatively concentrated, and the samples have better repeatability. PCoA obtained different contribution rates through different analysis methods, indicating that although the difference in microbial colonies between the two groups of samples was not significant, there were still certain differences.

**Figure 2:**
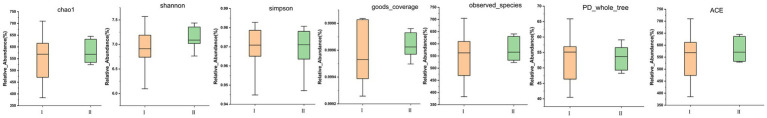
Effects of different diets on alpha diversity index of rumen microorganisms in Guizhou black male goats. **(A–E)** Represents the Chao1 index, Shannon index, Simpson index, goods_coverage index, and ACE index, respectively.

In the two diagrams of Circos (Figure 1D) and Phylogenetic (Figure 1E), the distribution of different ASVs of the three main dominant colonies at the phylum level could be visually observed. In the Phylogenetic diagram, we can also visually observe the evolutionary distance of different ASVs. From the Circos and Phylogenetic diagrams, it was concluded by that the detected rumen microorganism ASV in different test groups is mainly distributed in *Bacteroidota*. In the alpha_diversity analysis (Figure 2), *Chao1*, *Shannon*, *Simpson*, *goods_coverage*, observed-species, PD-whole-tree and *ACE* indexes were similar and did not reach a significant level between the two experimental groups (*p* > 0.05). The *goods_coverage* between the two groups was close to 1, indicating that the sequencing depth is reasonable. The species distribution is uniform and diverse with high reliability, covering all species, therefore, microbial sample data can be further analyzed.

**Figure 3:**
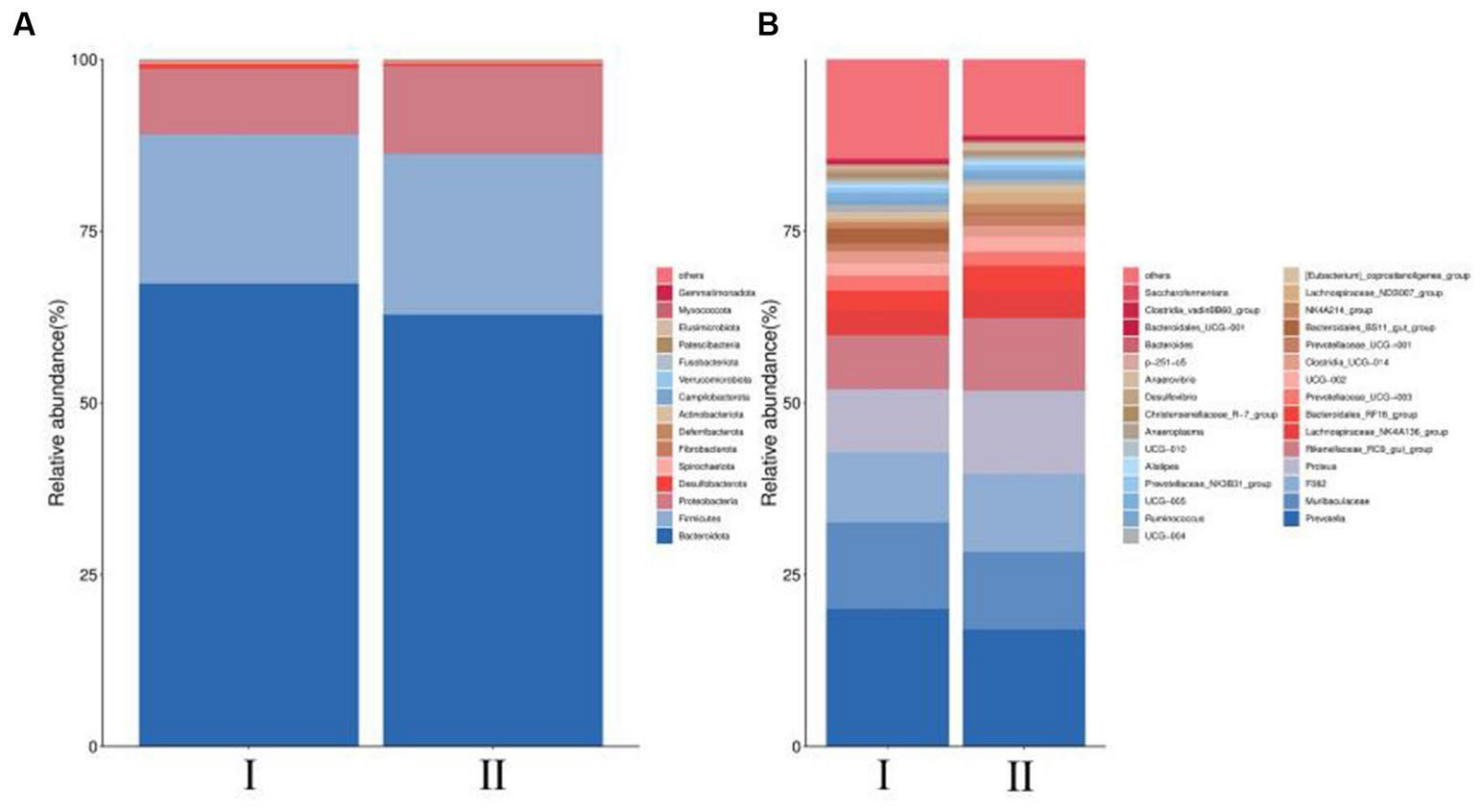
Effects of different diets on the phylum-level and genus-level colony abundance of rumen microorganisms in Guizhou black male goats. **(A)** Relative abundance of phylum horizontal species. **(B)** Relative abundance of genus horizontal species.

At the phylum level, the dominant bacteria are *Bacteroidota*, *Firmicutes* and *Proteobacteria* (Figure 3A). However, the differences in levels of *Bacteroidota*, *Firmicutes* and *Proteobacteria* were not significant (*p* > 0.05) (Table 6). At the genus level, *Prevotella*, *Muribaculaceae*, and *F082* were the main dominant genera (Figure 3B). The incorporation of 30% FVMR in Guizhou black male goats FTMR diets could change the colony composition of rumen microorganisms at the genus level (Table 7). The inclusion of 30% FVMR decreased *Bacteroidales_BS11_gut_group*, *Desulfovibrio*, and *Christensenellaceae_R-7_group* (*p* < 0.05), while increasing *Lachnospiraceae_ND3007_group* when compared to the control group (*p* < 0.01).

**Table 6:** Effects of different diets on the relative abundance of ruminal bacterial communities at the phylum level (average relative abundance ≥0.1% for at least one treatment) of Guizhou black male goats (%).

Item	Group		SEM	*p*-value
	I	II		
*Bacteroidota*	68.24	63.20	1.67	0.135
*Firmicutes*	21.12	23.24	1.02	0.309
*Proteobacteria*	9.29	12.55	0.94	0.083
*Desulfobacterota*	0.69	0.41	0.07	0.057
*Spirochaetota*	0.20	0.15	0.02	0.094
*Fibrobacterota*	0.12	0.13	0.03	0.787
*Deferribacterota*	0.11	0.10	0.01	0.648

I, Traditional FTMR without FVMR diets; II, 30% FVMR in FTMR diets.

**Table 7:** Effects of different diets on the relative abundance of ruminal bacterial communities at the genus level (average relative abundance ≥0.5% for at least one treatment) of Guizhou black male goats (%).

Phylum	Genus	Group		SEM	*p*-value
		I	II		
*Bacteroidota*	*Prevotella*	20.72	17.44	2.15	0.459
	*Muribaculaceae*	12.24	11.27	0.66	0.473
	*F082*	10.19	11.22	1.20	0.674
	*Rikenellaceae_RC9_gut_group*	7.65	10.56	0.90	0.107
	*Bacteroidales_RF16_group*	2.86	3.66	0.39	0.311
	*Prevotellaceae_UCG-003*	2.27	2.04	0.28	0.685
	*Prevotellaceae_UCG-001*	1.14	1.70	0.16	0.088
	*Bacteroidales_BS11_gut_group*	2.34^a^	0.20^b^	0.49	0.026
	*Prevotellaceae_NK3B31_group*	0.61	0.72	0.06	0.342
	*Alistipes*	0.52	0.57	0.04	0.590
Firmicutes	*Lachnospiraceae_NK4A136_group*	3.60	3.92	0.19	0.409
	*Clostridia_UCG-014*	1.71	1.61	0.14	0.738
	*Ruminococcaceae_NK4A214_group*	0.86	1.28	0.22	0.349
	*Ruminococcaceae_UCG_002*	1.72	2.20	0.30	0.443
	*Lachnospiraceae_ND3007_group*	0.47^b^	1.64^a^	0.20	<0.01
	*Eubacterium_coprostanoligenes_group*	0.97	1.08	0.11	0.602
	*Lachnospiraceae_UCG-004*	0.99	0.78	0.24	0.678
	*Ruminococcus*	0.80	0.83	0.11	0.912
	*Ruminococcaceae_UCG-005*	0.94	0.59	0.23	0.471
	*Lachnospiraceae_UCG-010*	0.57	0.49	0.07	0.568
	*Anaeroplasma*	0.51	0.53	0.14	0.957
	*Christensenellaceae_R-7_group*	0.60^a^	0.30^b^	0.07	0.024
*Proteobacteria*	*Proteus*	8.91	11.85	0.96	0.129
	*Desulfovibrio*	0.58^a^	0.32^b^	0.07	0.049
Other	*uncultured*	1.62	2.42	0.21	0.056
	*Other*	7.91	3.03	1.60	0.130

*p* < 0.05, significant; *p* < 0.01, extremely significant. SEM, standard error of the mean. Different superscript letters indicate significant differences.

I, Traditional FTMR without FVMR diets; II, 30% FVMR in FTMR diets.

**Figure 4:**
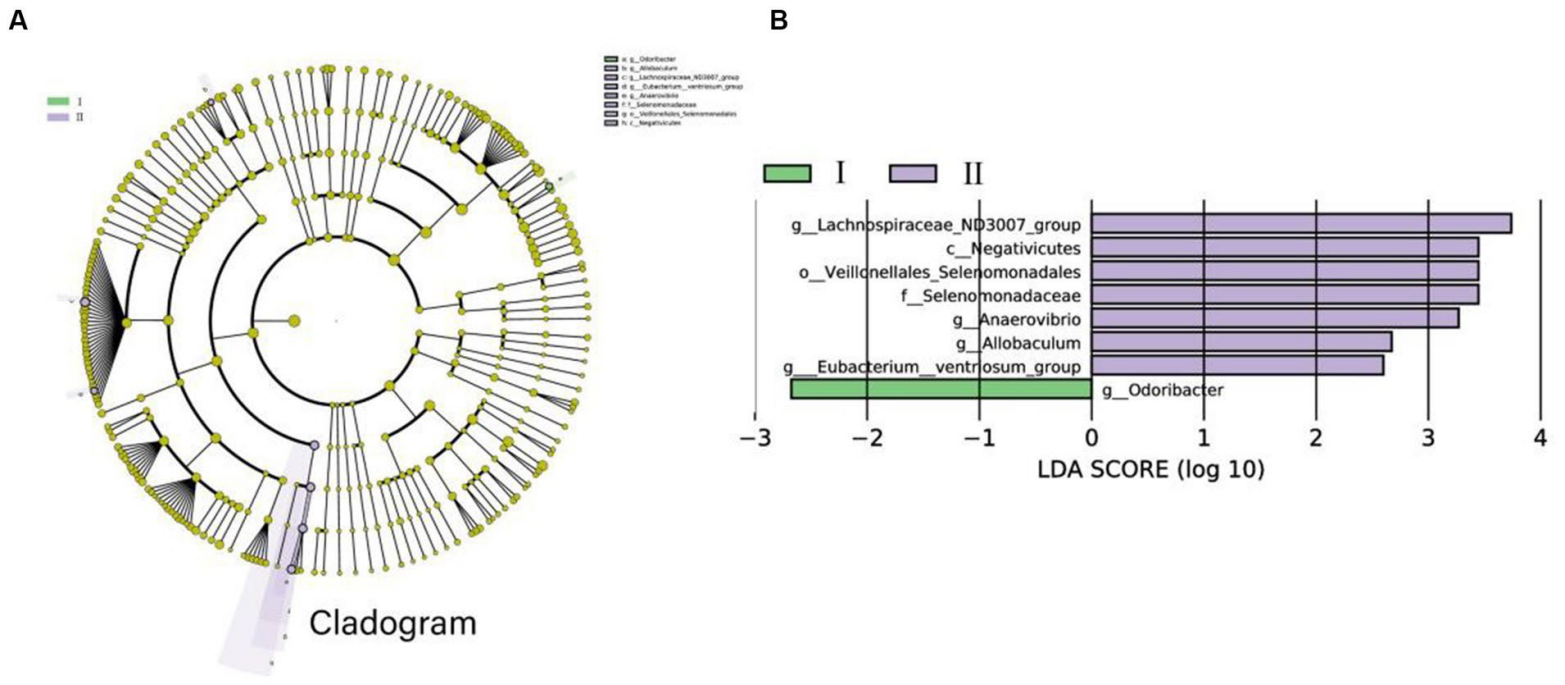
The microbiomes of the two groups were described using LEfSe and LDA analysis according to ASV differences. **(A)** The histogram of the distribution of LDA values was calculated with a score of LDA scores >2. The length of the bars represents the abundance of different species. **(B)** Example map of different species annotation branches in the figure; different colors indicate different groups. The yellow nodes represent the species with no significant difference between the two groups. The diameter of the node is proportional to the relative abundance. Each layer of nodes represents the phylum/class/order/family/genus from the inside to the outside and each layer of species. The marked annotations indicate the phylum/class/order/family/genus from the inside to the outside; the species names represented by English letters in the figure are displayed in the legend on the right.

All microbial data were used in the LEfSe analysis, which correctly identified the important bacterial groups connected to the two groups. Figure 4 depicts a representative structural cladogram of major microbiota showing the relative abundance of species within this group. From the Cladogram and LDA graphs, it can be found that there is 1 colony (*g_Odoribacter*) with high abundance in group I and 7 colonies (For example, g*_Lachnospiraceae_ND3007_group*, *g_Anaerovibrio*, and *g_Allobaculum*, etc.) with high abundance in group II (Figure 4).

### 3.5 Correlation analysis

**Figure 5:**
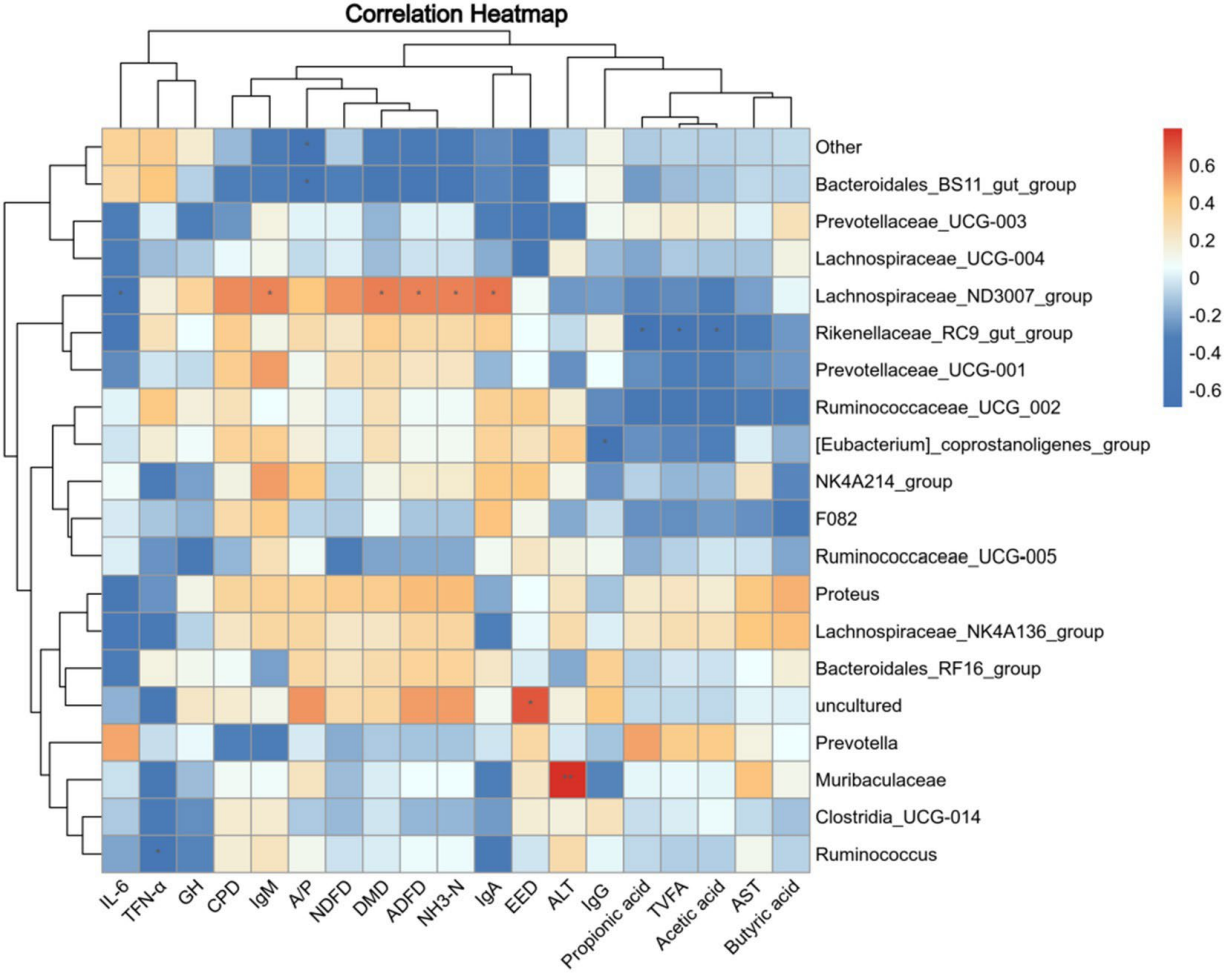
Correlation analysis of rumen fermentation parameters and nutrient digestibility with microbial abundance. TVFA, Total volatile fatty acids; EED, Apparent digestibility of crude fat; A/P, Acetic acid/propionic acid; CPD, Apparent digestibility of protein; NDFD, Apparent digestibility of neutral detergent fiber; DMD, Apparent digestibility of dry matter; ADFD, Apparent digestibility of acid detergent fiber. Red indicates a positive correlation; blue indicates a negative correlation. *p < 0.05.

Correlations between microbiome composition and rumen fermentation parameters and digestibility are shown in Figure 5. The relative abundance of *Rikenellaceae_RC9_gut_group* exhibited a negative correlation with acetic acid, TVFA, and propionic acid (*p* < 0.05). Similarly, the relative abundance of *F082*, *Ruminococcaceae_UCG_002*, *Prevotellaceae_UCG-001,* and *[Eubacterium]_coprostanoligenes_group* in the rumen were higher. In addition, the content of acetic acid, propionic acid, butyric acid, and TVFA will be reduced. The apparent digestibility of EE was positively correlated with the relative abundance of *uncultured* flora (*p* < 0.05) and negatively correlated with the relative abundance of *Bacteroidales_BS11_gut_group*, *Prevotellaceae_UCG-003,* and *Lachnospiraceae_UCG-004* (*p* > 0.05). The A/P ratio was shown to be negatively related to the relative abundance of *Bacteroidales_BS11_gut_group* and other genera (*p* < 0.05). The digestibility of DMD, IgA, IgM, NH_3_-N, and ADFD was positively correlated with the relative abundance of *Lachnospiraceae_ND3007_group* and negatively correlated with the relative abundance of *Bacteroidales_BS11_gut_group*. In addition, *[Eubacterium]_coprostanoligenes_group* relative abundance changes were negatively correlated with IgG, while *Ruminococcus* abundance changes were negatively correlated with TFN-α (*p* < 0.05). There is a positive correlation between the relative abundance of *Muribaculaceae* and changes in ALT (*p* < 0.05).

## 4 Discussion

### 4.1 Effects of growth performance and apparent digestibility

*Flammulina velutipes* mushroom residue has high moisture content and also high in cellulose, which easily contaminated by fungi and bacteria if not treated in time. It will begin to decompose after 2–3 days and produce harmful compounds after 1 week (Kim et al., 2007). The mushroom residue treated by microbial fermentation can prolong the shelf life and improve the feed’s palatability and nutritional value (Gao et al., 2008; Kwak et al., 2008). Although feed nutritional value and palatability data were not presented in this study, previous behavioral studies have confirmed that feeding FVFM in the FTMR could improve feed quality and palatability (Long et al., 2023b). In the breeding process, animal growth performance directly determines economic benefits to a large extent, so animal ADG and FCR are the key research indicators. Huang et al. (2021) found that the ADG of Liuyang black goats fed with 65% mixed concentrate +35% oyster mushroom chaff and whole plant rice co-fermented material increased by 18.33%, and FCR decreased to 5.20. In this study, the ADG of the Guizhou black male goats increased by 10.83% and the FCR was significantly lower than that of the control group. We also found that the cost of feeding the experimental group containing 30% FVMR in FTMR diets was 25% lower than that of the control group, and the economic benefit of each goat increased by 10.82%. The findings of this study agree with those of Huang et al. (2021) and Kang et al. (2022). However, it is inconsistent with the research results of Guo et al. (2015) that adding FVMR to the diet had no significant effect on the growth performance of Boer goats.

Feed digestibility is positively correlated with the absorption rate of nutrients absorbed by animals and the quality of feed, which was very important for the growth and development of ruminants (Zhang et al., 2022). Relevant research has demonstrated that fermenting feed could increase the utilization rate of feed (Kawamoto et al., 2009) and the incorporation of white-rot fungi and enzymatic bacteria in diets could improve feed quality and digestibility (So et al., 2020; Clark et al., 2022). In this study, the incorporation of 30% FVMR in diets of Guizhou black male goats could increase the apparent digestibility of DM, CP, and NDF. The reason may be that the nutritional properties and special composition of the feed raw materials (bacteria residue) themselves may directly increase the apparent digestibility (Li et al., 2015; Meng et al., 2016). Moreover, we have adopted different treatment methods, adding white-rot fungi, mixed starter culture and enzyme preparation to the FVMR feed, which will also greatly improve the apparent digestibility of the feed. When animals ingest feed to reach the energy requirements, DMI intake of feed will be reduced, and because the moisture content of FVFM feed after fermentation was higher than that of the control group, it may also be the reason for reducing DMI intake of feed. Ultimately, the FCR decreases.

### 4.2 Effects of rumen fermentation

The fluctuation of rumen pH is the most intuitive indicator reflecting the state of rumen fermentation and internal environment stability, which normal range is 6.0 ~ 7.0 (Guo et al., 2022). Although the rumen pH value of the FVMR in the FTMR diet was higher than that of the control group, both groups were within the normal range. Therefore, it could be considered that feeding Guizhou black male goats with FVFM in the FTMR would not cause ruminal acidosis.

Acetic acid and butyric acid can be converted into each other in the rumen (Wang et al., 2020). 28% of acetic acid is not absorbed by the rumen in the form of acetic acid, but acetic acid could react with acetyl-CoA or butyryl-CoA transferase to produce butyric acid, which is then absorbed by rumen microorganisms (Kristensen, 2001; Hackmann and Firkins, 2015). In this study, we concluded that the acetic acid content of Guizhou black male goats fed 30% FVFM in the FTMR was significantly lower than that of the control group, while the contents of propionic acid and butyric acid tended to increase. It might be because FVFM in the FTMR could improve the activity of acetyl-CoA and butyryl CoA, and accelerate the conversion between acetic acid and butyric acid (Henderson et al., 2015). However, goats may promote microbial metabolism through an energy dissipation process that continuously converts acetic acid to butyric acid. Additionally, FVFM in the FTMR might also change the rumen fermentation mode from acetic acid to propionic acid (Chen et al., 2021), this model needs to be further confirmed under the conditions of this study. Finally, the content of acetic acid and the ratio of acetic acid to propionic acid is reduced. In addition, butyric acid could usually participate in the development of rumen papilla by stimulating the metabolism of rumen epithelial cells (Poudel et al., 2019) and mutton sheep mainly use propionic acid produced from sugar and starch fermentation to produce glucose through gluconeogenesis to provide more energy for the body (Astawa et al., 2011; Gunun et al., 2018; Zhang et al., 2022). Thus, while propionic acid and butyric acid were conducive to improving animal growth, which could explain the higher growth performance obtained in this study.

### 4.3 Effects of serum biochemical indicators

Serum biochemical indicators are affected by factors such as animal species, species, age, sex, dietary nutritional structure, and seasonal climate changes, which could reflect the body’s health, body nutrition level, and metabolic state (Piccione et al., 2010). As one of the peptide hormones, GH could participate in the regulation of animal reproductive function and promote muscle development, but to a certain extent, it could also promote the differentiation, proliferation, and migration of some cancer cells (Imbesi et al., 2014). This study found that feeding 30%, FVMR FTMR can significantly increase goats’ GH value and better promote goats’ growth and development. IgA, IgG, and IgM produced in the body’s first immune response are important immunoglobulins of the body and are the main antibodies of the body’s second immune response to pathogens (Çölkesen et al., 2022). This study found that 30% FVMR in FTMR diets increased the levels of IgA and IgM in goat serum, which indicates that feeding 30% FVMR in FTMR diets improved the immune capacity of goats. The IL-6 anti-inflammatory factor secreted by Th2 cells can produce a large amount of immunoglobulin by stimulating B cells, which have anti-infective, inhibit, and kill tumor cells (Calleja-Agius and Brincat, 2008; Wu et al., 2011). However, we have failed to identify the specific reasons and mechanisms of IL-6 in this study was lower than those in the control group, which needs further study. AST and ALT have the function of catalyzing the conversion of amino acids into keto acids and are considered to be crucial transaminase enzymes in various biological processes (Yang et al., 2022). Under normal circumstances, the activity of ALT and AST in the body’s serum is low. If the liver tissue cells are damaged, lesions occur, and the function is impaired, the body’s AST and ALT will enter the blood to increase the activity of ALT and AST in the serum (Akrami et al., 2015; Tan et al., 2016). In this study, it was found that feeding 30% FVMR in FTMR diets decreased the content of ALT and AST. It shows that feeding 30% FVMR in FTMR diets could improve the immune ability of Guizhou black male goats and maintain liver health.

### 4.4 Effects of rumen microflora

Rumen microorganisms comprise three major groups of *protozoa*, *bacteria,* and *fungi*. The unique existence of these microorganism groups has created a powerful digestive system for ruminants (Biscarini et al., 2018). Regulating the rumen microbial ecosystem to improve rumen fermentation in ruminants, improving animal productivity, and economic benefits have always been among the leading research hotspot in animal nutrition research (Patra and Saxena, 2011). The change in rumen microbial diversity has a key relationship with the diet structure. By adjusting the diet structure, the rumen microbial diversity can be improved, and the health of ruminants can be guaranteed (Henderson et al., 2015). At the phylum level, the proportion of *Bacteroidetes* and *Firmicutes* accounted for more than 70% of rumen microorganisms, and they were the dominant colonies of rumen microorganisms in ruminants (Singh et al., 2012; Jami et al., 2013; Liu et al., 2017). This study also obtained consistent results. The sum of *Bacteroidetes* and *Firmicutes* colonies between the two groups accounted for more than 70% of the rumen microorganisms. *Bacteroidetes* in the rumen are an essential flora that promotes the degradation of polysaccharides, proteins, and carbohydrates in feed in ruminants (Naas et al., 2014); it can degrade high-molecular organic matter and improve the innate immune response by enhancing the intestinal mucosal barrier function (Thomas et al., 2011; Magrone and Jirillo, 2013). *Bacteroidetes* in the rumen are crucial for the synthesis of acetate and propionate as well as the degradation of non-cellulosic plant-based substances (Söllinger et al., 2018). This is the main reason that the proportion of acetic acid and propionic acid in the rumen of the control group is higher than that of the experimental group II. *Firmicutes* are related to energy metabolism, and they carry many genetic codes that can produce a variety of digestive enzymes to decompose various nutrients; they are the most critical bacterial group for ruminants to absorb protein and improve fiber utilization (Kaakoush and Nadeem, 2015; Petri et al., 2019). *Proteobacteria* are mainly Gram-negative bacteria, including, *Escherichia coli*, *Salmonella,* and other pathogenic bacteria, which can degrade soluble carbohydrates. When the content is higher than 19%, indicating that the rumen microbial ecosystem is unstable (Auffret et al., 2017). In this study, the content of *Proteobacteria* in the two test groups was lower than 19%, and the rumen microbial ecosystem was stable. It shows that feeding 30% FVMR in FTMR diets does not affect the stability of the rumen microbial system of black goats. In addition, the relative abundance of Bacteroidetes in group II was lower than that in the control group, but the relative abundance of Firmicutes was higher than that in the control group. This provides certain evidence that FVMR can improve the ruminal microbial composition of Guizhou black male goats.

Generally, the most dominant genus in the rumen of ruminants is *Prevotella* (Bowen et al., 2018). Its primary function is to degrade cellulose, starch, hemicellulose, and protein (Li and Guan, 2017). *Prevotella* was positively correlated with the proportion of acetic acid but negatively correlated with the body’s MCP concentration, gas production, and the proportion of valerate and butyrate (Zhou et al., 2022). The reason may be that why the acetic acid in the group II was lower than that in the control group, but the butyric acid was higher than that in the control group. *Muribaculaceae* belongs to *Bacteroidetes*, which is abundant in the intestines of mice. This genus is a newly identified genus name. There is still a lack of in-depth understanding of its specific functions. The changes in its abundance are mainly related to the host and its dietary conditions (Zhou et al., 2020). In this study, because both *Muribaculaceae* and *Prevotella* belong to *Bacteroidota*, we believe that the function of *Muribaculaceae* may be related to the degradation of cellulose, starch, and protein, and further research is needed to prove it. *Rikenellaceae_RC9_gut_group* and *Ruminococcus* are the main bacteria of rumen microorganisms, which can secrete a large amount of oligosaccharase, cellulase, and hemicellulase and participate in the decomposition and absorption of protein and carbohydrates (Dai et al., 2015; Zhu et al., 2021). Therefore, it promoted fiber digestion in test group II more than in control. The relative abundance of *Prevotellaceae_UCG-003* was significantly positively correlated with the valeric acid and propionic acid ratio, and there was a promoting effect in the process of polysaccharide degradation (Flint et al., 2008). This may also lead to lower propionic and valeric acid in group II in this study than in the control group. The change of rumen microbes has an important relationship with the body’s immune regulation, disease resistance, and animal growth (Wang et al., 2021). *Lachnospiraceae_NK4A136_group* and *Lachnospiraceae_ND3007_group* belong to the *Lachnospiraceae* family, and many microorganisms can produce butyric acid, which can inhibit inflammation and enhance the integrity of the epithelial barrier and are positively correlated with IgG and IgM (Meehan and Beiko, 2014; Sarmikasoglou and Faciola, 2022). In addition, *Ruminococcus* may inhibit inflammation and promote growth in animals through butyrate production (Scaldaferri et al., 2016). Relevant studies have shown that *Fibrobacter* and *Prevotellaceae UCG-003* negatively correlate with enhancing immune function (Wang et al., 2021). In this study, Feeding 30% FVMR in FTMR diets could improve the rumen microbial composition of Guizhou black male goats.

## 5 Conclusion

In the context of this study, we attempted to unravel the effects of the FVMR diet on the fattening effect and rumen health of Guizhou black male goats. Our findings demonstrated that 30% FVMR in Guizhou black male goats FTMR diets could improve rumen microbial composition and reduce the content of acetic acid. Moreover, the apparent digestibility of DM, CP, and NDF is improved, it also could improve the immunity. Finally, 30% FVMR in FTMR diets increased ADG and decreased FCR of Guizhou black male goats. Consequently, the feed cost is 25% lower than that of the control group, and the average profit per sheep is 17.35 CNY higher than that of the control group. In conclusion, 30% FVFM in the FTMR to feed Guizhou black male goats is an effective and promising method to reduce feeding costs and improve the economic benefits of Guizhou black male goats. Notably, We believe that many microorganisms in the rumen after feeding FVMR are closely related to the immune regulation and health of the animal body, and the specific influencing mechanisms and functions require further research and verification.

## Data availability statement

The data presented in the study are deposited in the NCBI repository, accession number PRJNA1063023.

## Ethics statement

All animals were meticulously conducted in accordance with animal welfare guidelines and were subject to stringent regulatory oversight by the Experimental Animal Ethics Committee of Guizhou University in Guizhou, China (EAE-GZU-2021-E024). The study was conducted in accordance with the local legislation and institutional requirements.

## Author contributions

YL: Writing – original draft, Writing – review & editing. WX: Data curation, Methodology, Writing – review & editing. YZ: Data curation, Methodology, Writing – review & editing. CY: Investigation, Software, Writing – review & editing. DW: Data curation, Supervision, Writing – review & editing. YY: Data curation, Supervision, Writing – review & editing. CS: Data curation, Supervision, Writing – review & editing. PP: Project administration, Resources, Visualization, Writing – review & editing. YH: Data curation, Funding acquisition, Investigation, Project administration, Resources, Supervision, Writing – review & editing, Writing – original draft.
