# Correction: Effects of *Flammulina velutipes* mushroom residues on growth performance, apparent digestibility, serum biochemical indicators, rumen fermentation and microbial of Guizhou black goat

**DOI:** 10.3389/fmicb.2026.1790100

**Published:** 2026-02-11

**Authors:** Yong Long, Wen Xiao, Yanpin Zhao, Chao Yuan, Defeng Wang, Yang Yang, Chaozhi Su, Pramote Paengkoum, Yong Han

**Affiliations:** 1 Guizhou University of Traditional Chinese Medicine, Guiyang, China; 2 Institute of Animal Husbandry and Veterinary Sciences, Guizhou Academy of Agricultural Sciences, Guiyang, China; 3 Key Laboratory of Animal Genetics, Breeding and Reproduction in the Plateau Mountainous Region, Ministry of Education, Guizhou University, Guiyang, China; 4 School of Animal Technology and Innovation, Institute of Agricultural Technology, Suranaree University of Technology, Nakhon Ratchasima, Thailand

**Keywords:** apparent digestibility, growth performance, rumen fermentation, rumen microorganisms, serum biochemical indicators

There was a mistake in Figure 1 as published. The authors mistakenly used an image from another experiment showing PCoA and NMDS analyses in 11 replicates (*n* = 11) for each treatment group. The correct Figure 1 appears below.

**Figure 1:**
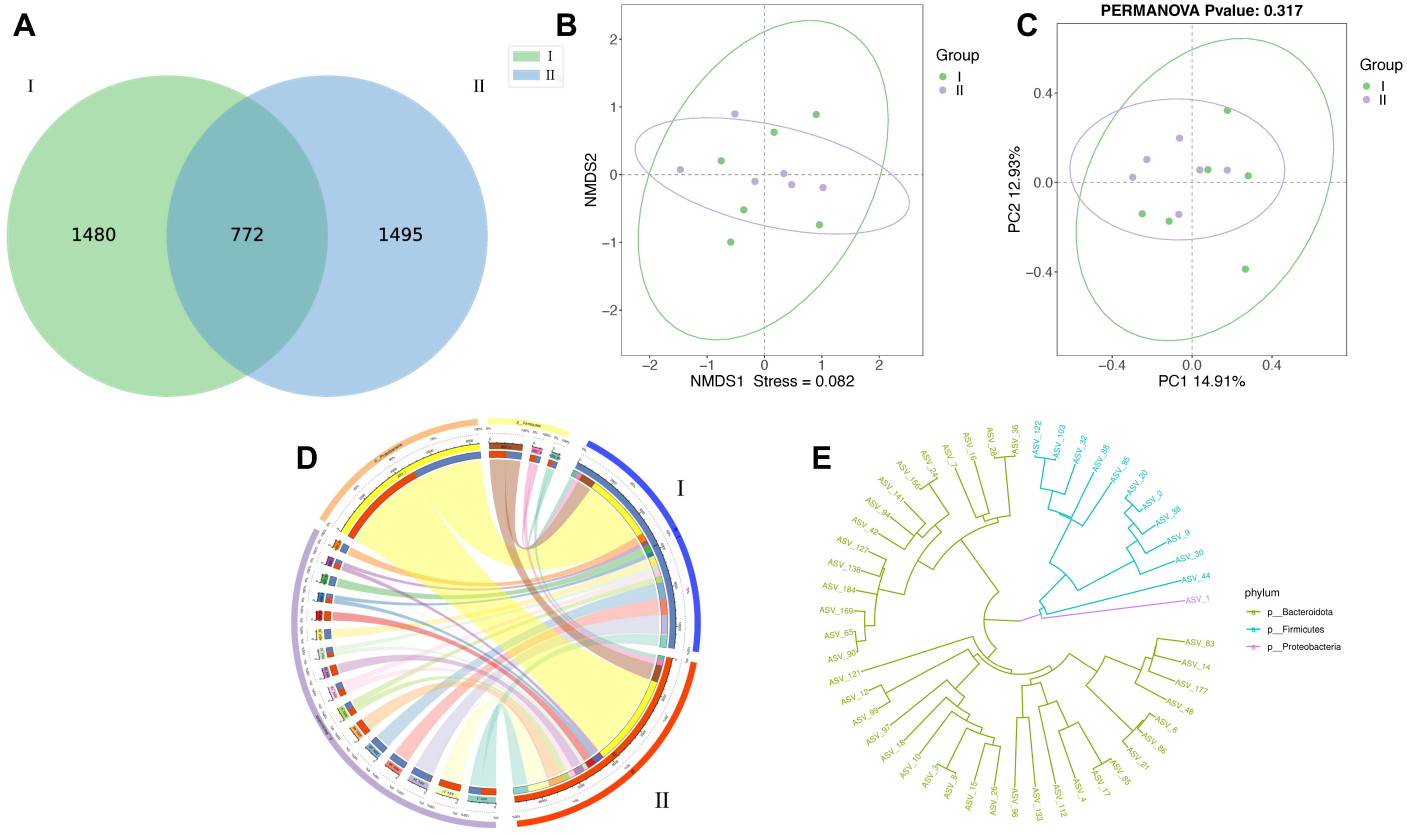
Effects of different diets on rumen microorganisms of Guizhou black male goats. **(A–E)** Are ASV-Venn, Principal coordinate analysis (PCoA) non-metric multidimensional scale analysis (NMDS), ASV-Circos, and ASV- Phylogenetic diagram analysis of I and II.

The details of the repetition count for the rumen microbial analysis were omitted from the Materials and Methods section. The first sentence in Section 2.5 *Rumen fluid collection, rumen fermentation parameters, and rumen microbial testing*, paragraph one has been edited as follows:

**Sentence as published:** On the morning of the end of the experiment, 2 h after the morning feeding, the rumen fluid was collected with a negative pressure collector, and the catheter was inserted into the oral esophagus until the middle of the rumen.

**Revised Sentence:** On the morning of the last day of the experiment, 2 h after feeding, six goats were randomly selected from the 11 goats in each group to collect rumen fluid using a negative-pressure sampler, with the tube inserted orally via the esophagus into the mid-rumen.

The original version of this article has been updated.

